# Childhood risk factors for substance abuse in a clinical sample of adults with attention-deficit / hyperactivity disorder (ADHD) symptoms in an addiction outpatient clinic

**DOI:** 10.1192/j.eurpsy.2021.1537

**Published:** 2021-08-13

**Authors:** M. Laizāne, M. Ennītis, N. Bezborodovs, I. Landsmane

**Affiliations:** 1 Faculty Of Residency, Riga Stradins University, Riga, Latvia; 2 Department Of Psychiatry And Narcology, Riga Stradins University, Riga, Latvia; 3 Department Of Addiction Medicine, Riga Psychiatry and Narcology Centre, Riga, Latvia

**Keywords:** Substance Use Disorder, Attention-deficit / Hyperactivity disorder (ADHD)

## Abstract

**Introduction:**

Substance use disorder (SUD) in patients with ADHD symptoms is associated with a poorer treatment prognosis. The study is aimed to investigate psychosocial risks factors for developing SUD in patients with ADHD.

**Objectives:**

To examine the associations between self-reported weak academic performance, repetition of a grade, single – parent family, self-reported quality of parent – child relationship, conduct problems in childhood and SUD in adulthood in a sample of outpatients with ADHD symptoms of an addiction medicine clinic in Riga, Latvia.

**Methods:**

Self-report surveys, containing Adult ADHD Self-Report Scale (ASRS-v I.I), were completed by outpatients of addiction clinic, including healthy control subjects (adults without addiction, formally assessed for fitness to drive, firearms licensing etc.). Patients then were examined in relation to childhood risk factors.

**Results:**

Survey was compleated by 341 outpatients – 98 (28,7%) healthy controls and 243 (71,3%) patients with SUD (mean age, 36,8 and 37,7, respectively; 76,4% males). 62 (18,1%) patients were tested positive for ADHD, of whom 12 (19,4%) were healthy subjects and 50 (80,6%) were with SUD. Data shows connection between conduct problems in childhood (p=0,010), single - parent family (p=0,010), repetition of a grade (p=0,026) and SUD in adults with ADHD symptoms. Comparing patients with and without ADHD symptoms, there was found no significant association between these factors, except for conduct problems (p=0,015).

**Conclusions:**

The study found preliminary evidence that adults with ADHD symptoms who experienced single – parenthood in childhood, had conduct problems or repeated a grade are more likely to develop SUD. Futher investigation is required.

